# Carbon Nanotubes Based 3-D Matrix for Enabling Three-Dimensional Nano-Magneto-Electronics

**DOI:** 10.1371/journal.pone.0040554

**Published:** 2012-07-12

**Authors:** Jeongmin Hong, Eugenia Stefanescu, Ping Liang, Nikhil Joshi, Song Xue, Dmitri Litvinov, Sakhrat Khizroev

**Affiliations:** 1 Department of Electrical and Computer Engineering, Florida International University, Miami, Florida, United States of America; 2 Department of Electrical Engineering, University of California Riverside, Riverside, California, United States of America; 3 Seagate Technology, Bloomington, Minnesota, United States of America; 4 MultiDimension, San Jose, California, United States of America; 5 Department of Electrical Engineering, University of Houston, Houston, Texas, United States of America; Queen’s University at Kingston, Canada

## Abstract

This letter describes the use of vertically aligned carbon nanotubes (CNT)-based arrays with estimated 2-nm thick cobalt (Co) nanoparticles deposited inside individual tubes to unravel the possibility of using the unique templates for ultra-high-density low-energy 3-D nano-magneto-electronic devices. The presence of oriented 2-nm thick Co layers within individual nanotubes in the CNT-based 3-D matrix is confirmed through VSM measurements as well as an energy-dispersive X-ray spectroscopy (EDS).

## Introduction

The progress of planar silicon-based electronics technology – described by Moore’s Law – is coming to an end. [1], [2] Gate-length scaling has pushed the gate-dielectric and junction technology to its physical limits and has resulted in a design in which billions of transistors are interconnected by tens of kilometers of wires packed into an area of square centimeters - new materials and processes have been introduced, but it is expected that the continued increase in interconnect wiring will ultimately lead to the demise of further CMOS scaling, quite apart from the very high power and associated thermal budgets that are required to drive such an architecture. [3] The electronics industry will be forced to meet the demand for additional functionality in a reduced footprint with revolutionary new electronic devices based on new state variable rather than electronic motion and by the extension of electronics into the third dimension. These next generation technologies will extend Moore’s Law and create dynasties of low power electronics with greater capabilities at a fraction of the cost.

The leap in the integration of devices can be addressed by 3-D technology. [4] Conventional electronics is based on 2-D planar processes, but this is becoming prohibitively expensive as well as a barrier to performance. By stacking devices and interconnecting them in a 3-D arrangement, a huge leap in functional density is possible together with reduced power consumption. 3-D integration is a cornerstone of the coming revolution in electronics not only because of the possibility of high-density architectures but also because it will enable the introduction of disparate signals and new materials and devices. [5]–[11] The 3-D device architecture allows functionally integrating different materials in one system thus providing a platform to combine the advantages of nanomagnetic structures with the unprecedented electronic and thermal properties of new materials such as graphene and other Carbon based nano-formulations. Using a 3-D matrix of nanomagnetic nodes “connected” via magnetic fields promises also to substantially reduce the number of physical wires and interconnects (vias) which is another important roadblock in the current 2-D planar VLSI technology. Nanomagnetic devices can be partially polarized and therefore naturally allow for multi-valued signal coding. In nanomagnetic devices, multi-valued signals can be reliably achieved with high noise tolerance, no increased power consumption, and no significantly increased circuit complexity, thus making multi-valued logic a cost effective and power efficient signal processing solution, further increasing the computing capacity per unit area or volume.

**Figure 1:**
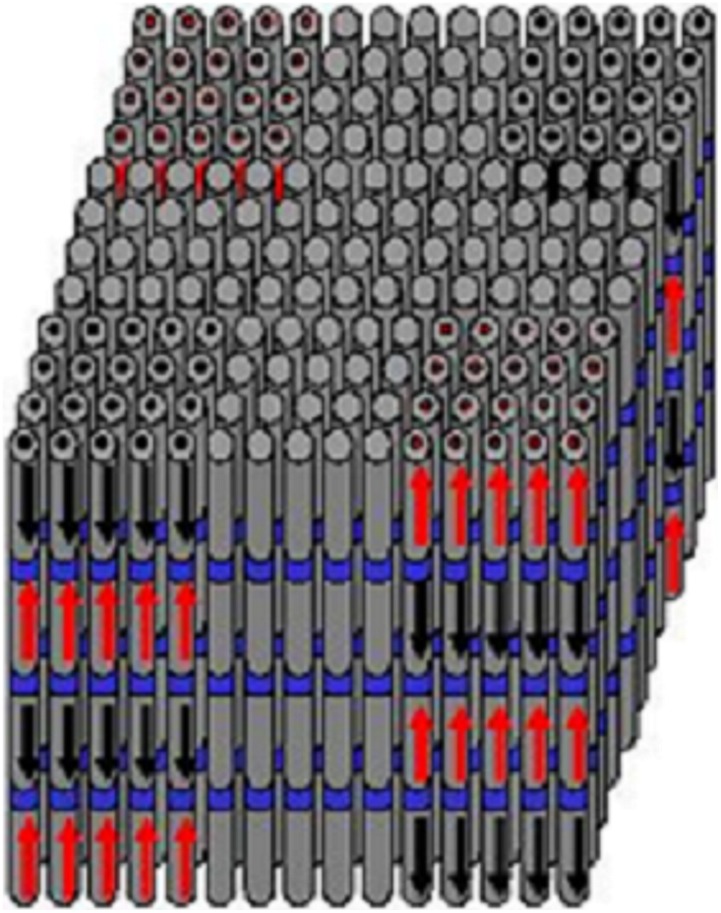
An array of vertically aligned CNTs with various magnetic materials deposited inside individual tubes. The magnetic anisotropy is shape-induced.

One of the innovative features of densely packed vertically aligned CNTs is the potential to be used as the 3-D matrices (templates) for enabling next-generation 3-D nano-magnetoelectronic devices. CNTs have been a promising candidate to build future electronic devices due to their many attractive properties. [12]–[19] In this paper, as a trivial example, a CNT matrix is used to control magnetic properties in nano scale.

## Results and Discussion

The inherent to the 3-D-matrix shape-induced magnetic anisotropy (due to the vertically aligned CNTs) ideally allows using any magnetic material in a specific orientation. The significance of this feature can be illustrated on the example of perpendicular magnetic recording (PMR) currently used as the core technology in magnetic disk drives. [20] The recording media used in PMR has an intrinsically induced anisotropy and consequently is severely material limited. Indeed, there are only a handful of materials used in the industry such as a hexagonal closed packed (hcc) phase of Co-based compounds, Co/Pt (or Pd) multilayer, high anisotropy L1_0_ materials, and a few others. As a result, the optimization of magnetic properties of the recording media is challenging. On the contrary, with the use of CNT-based templates, ideally any magnetic material could be used with an effectively perpendicular orientation, as illustrated in Figure 1. Here, we show that even a magnetically “soft” material could be made relatively “hard” and oriented through inducing shape anisotropy. In this case, the saturation moment is defined by the material, while the anisotropy is due to the 3-D matrix architecture. More specifically, the shape-induced anisotropy can be controlled by controlling the size of the filled (by the magnetic materials) nanotubes, i.e. a longer nanotube would induce a stronger out-of-plane anisotropy.

**Figure 2:**
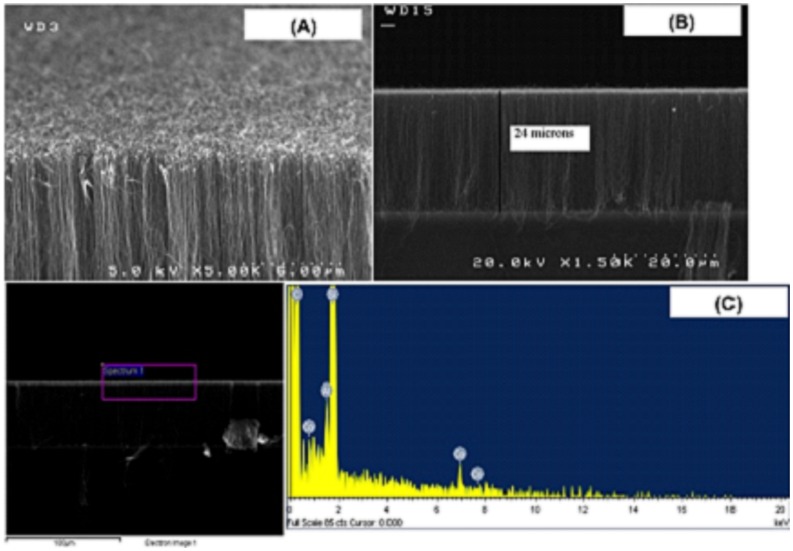
SEM images of (A) a vertically aligned CNT array and (B) a cross sectional image of an aligned CNT. (C) An energy-dispersive X-ray spectroscopy (EDS) of the composition of a vertically aligned CNT array. (The bottom left shows the targeted region for EDS pattern.).

There are many ways to deposit magnetic materials inside CNTs: 1) sputtering, 2) evaporation, 3) electroplating, and many others. In this particular experiment, we used porous Aluminum oxide (AAO) membranes to deposit a cobalt (Co) particles as a patterned array of catalyst sites. [21], [22] With such templates, with narrow distributions of diameters, Co nanoparticles were electrodeposited at the bottoms of openings to serve as catalysts in the subsequent pyrolysis step. Uniformly aligned carbon nanotubes were generated inside the pores of AAO through the catalytic pyrolysis of a hydrocarbon such as acetylene. The formed tubes had outer diameters equal to the inner diameters of the base pores. The desired cobalt particles were deposited within the nanotubes through the electroless deposition. Crystallite sizes in the range of 1.4 to 11.9 nm could be achieved. [23] Previous studies had shown that fcc, instead of hcp, was the stable and preferable structure of Co particles. [24], [25] It was found in some cases, Co would get inside the tubes and could occupy from 0 to 100 percent of a tube. Figure 2(A) and (B) represent SEM images of a vertically aligned high-aspect ratio CNT array. An energy-dispersive X-ray spectroscopy (EDS) of the composition of a vertically aligned CNT array is shown in Figure 2(C). The presence of Co is obvious from the spectrum.

**Figure 3:**
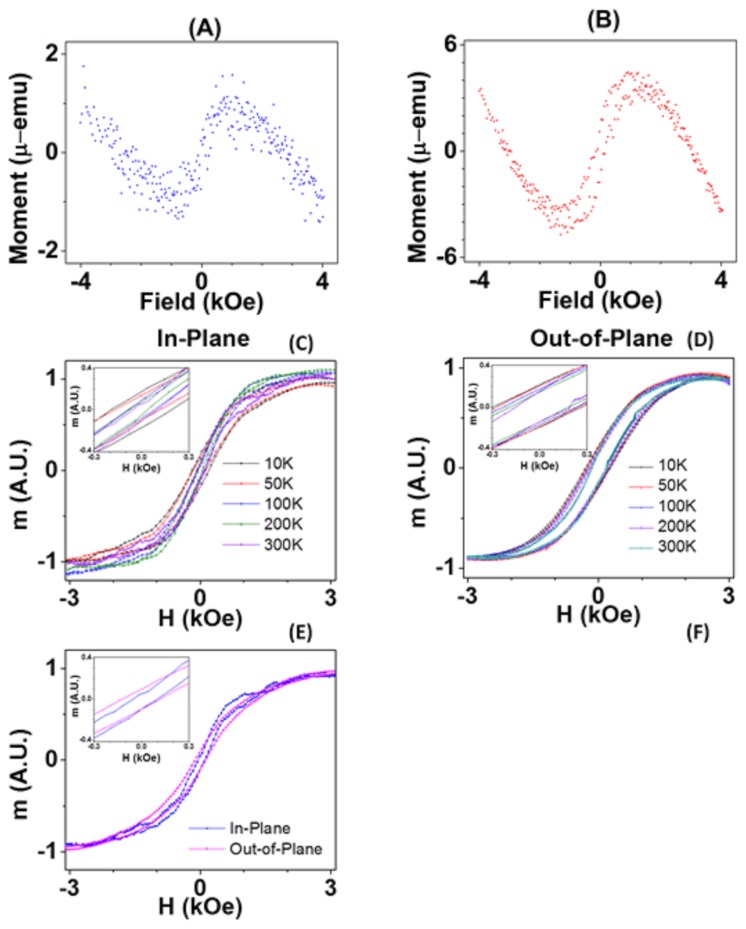
Magnetic moment (m) versus the applied field (H) hysteresis loops (A) along and (B) perpendicular to the plane directions at room temperature before subtracting diamagnetic background effects (originating from the substrates). (C) In-plane and (D) out-of-plane m-H loops for a range of temperature from 10 to 300 K after subtracting the diamagnetic background, (E) Room temperature m-H loops with out-of-plane and in-plane orientation, and (F) m-H loops from two directions before normalizing the magnetic moments. All the saturation values are above the value of the bulk fcc Co material [166 emu/g or 175 emu/g]. [27]–[29]

The magnetic moment of the CNT-based magnetic arrays was measured via vibrating sample magnetometry (VSM) PPMS (by Quantum Design, San Diego, CA). The measurements in two different directions, along and perpendicular to the plane are shown in Figures 3a and b, respectively. The more open hysteresis in the perpendicular direction and the anisotropy field higher than 1000 Oe (that is comparable to the saturation magnetization 4πM_s_ of the order of 1000 emu/cc) indicate the orientation of the magnetization is tilted toward the perpendicular direction and is shape induced. For comparison, the magneto-crystalline anisotropy of Co doesn’t exceed approximately 300 Oe, that is substantially smaller than the observed anisotropy value. Here, it should be mentioned that in the absence of CNT-tubes, the magnetization of Co would be in fcc (cubic) phase and preferentially in a plane direction.

Assuming that the anisotropy is indeed shape induced, i.e. 2πM_s_ (assuming the cylindrical shape of a nanotube) one can estimate that the approximate volume of Co in the CNT sample is of the order 2×10^−6^ emu/10^3^ emu/cc = 2×10^−9^ cc, which corresponds to the effective thickness of 2×10^−9^ cc/0.09 cm^2^∼2×10^−7^ cm = 2 nm. [26]

In summary, using an example of Co particles deposited inside individual CNTs, the study showed that densely packed vertically aligned CNTs could be indeed used a 3-D matrix for controlling the anisotropy direction of magnetic materials. The presence of oriented 2-nm thick Co layers within individual nanotubes in the CNT-based 3-D matrix was confirmed through VSM measurements as well as through an energy-dispersive X-ray spectroscopy (EDS).

## Materials and Methods

### Deposition of Cobalt Nanoparticles

Porous Aluminum oxide (AAO) membranes were used to pre-deposit cobalt (Co) particles as a patterned array of catalyst sites. Uniformly aligned carbon nanotubes were generated inside the pores. The desired cobalt nanostructures were filled in the interior of the nanotubes using electroless deposition.

### Vibrating Sample Magnetometry

M-H loop measurements were performed using the VSM option of a Quantum Design cryogenic physical property measurement system (PPMS) with a 9-Tesla superconducting magnet. Samples were mounted on a quartz paddle with regular disk holders, using GE-7031 varnish to withstand thermal cycling. To optimize the touchdown process, the samples were mounted with an upward offset of 35 mm.

### SEM and EDS Measurements

The SEM and EDS study was performed with JEOL JSM 6330F.

## References

[1] Wolf SA, Awschalom DD, Buhrman RA, Daughton JM, von Molnar S (2001). Spintronics: a spin-based electronics vision for the future.. Science.

[2] Tummala R (2006). Moore’s law meets its match.. IEEE Spectrum.

[3] Tanaka N, Yoshimira Y, Naito T, Miyazaki C, Nemoto Y (2005). Ultra-thin 3D-stacked SIP formed using room-temperature bonding between stacked chips.. in Proceedings of the 55^th^ Electronic Components and Technology Conference, Lake Buena Vista, FL, USA.

[4] Emma PG, Kursun E (2008). Is 3D chip technology the next growth engine for performance improvement? IBM J. Res. & Dev..

[5] Shen L, Chen W, Hung Y, Yang T, Leu F (2008). A scheme of array memory stacking to the multi-channel solid state disk (SSD) applications: high speed, high reliability, and green compliance.. in Proceedings of the 58^th^ Electronic Components and Technology Conference, Lake Buena Vista, FL, USA.

[6] Newman M, Muthukumar S, Schuelein M, Dambrauskas T, Dunaway P (2006). Fabrication and electrical characterization of 3D vertical interconnects.. in Proceedings of the 56^th^ Electronic Components and Technology Conference, San Diego, CA, USA.

[7] Ranganathan N, Prasad K, Balasubramanian N, Pey KL (2008). A study of thermo-mechanical stress and its impact on through-silicon vias. J. Micromech. Microeng..

[8] Lu KH, Ryu S, Zhao Q, Zhang X, Im J (2010). Thermal stress induced delamination of through silicon vias in 3-D interconnects.. in Proceedings of the 60^th^ Electronic Components and Technology Conference, Las Vegas, NV, USA.

[9] Ramaswami S, Dukovic J, Eaton B, Pamarthy S, Bhatnagar A (2009). Process Integration Considerations for 300 mm TSV Manufacturing. IEEE Trans. Dev. Mater. Reliab..

[10] Kikuchi H, Yamada Y, Ali A, Liang J, Fukushima T (2008). Tungsten through-silicon via technology for three-dimensional LSIs. Jpn. J. Appl. Phys..

[11] Rimskog M (2007). Through wafer via technology for MEMS and 3D integration.. in Proceedings of the 32^nd^ IEEE/CPMT International Electronics Manufacturing Technology Symposium, San Jose, CA, USA.

[12] Falvo MR, Clary GJ, Taylor RM, Chi V, Brooks FP (1997). Bending and buckling of carbon nanotubes under large strain.. Nature.

[13] Jiang H, Liu B, Huang Y, Hwang KC (2004). Thermal expansion of single wall carbon nanotubes. J. Eng. Mater. Technol..

[14] Wei BQ, Vajtai R, Ajayan PM (2001). Reliability and current carrying capacity of carbon nanotubes. Appl. Phys. Lett..

[15] Collins PG, Hersam M, Arnold M, Martel R, Avouris Ph (2001). Current saturation and electrical breakdown in multiwalled carbon nanotubes. Phys. Rev. Lett..

[16] Ragab T, Basaran C (2009). Joule heating in single-walled carbon nanotubes. J. Appl. Phys..

[17] Hone J, Whitney M, Piskoti C, Zettl A (1999). Thermal conductivity of single-walled carbon nanotubes. Phys. Rev.. B.

[18] Bom D, Andrews R, Jacques D, Anthony J, Chen B (2002). Thermogravimetric analysis of the oxidation of multi-walled carbon nanotubes: evidence for the role of defect sites in carbon nanotube chemistry. Nanolett..

[19] Awano Y, Sato S, Nihei M, Sakai T, Ohno Y (2011). Carbon nanotubes for VLSI, interconnect and transistor applications. Proc.. IEEE.

[20] Iwasaki S, Nakamura Y (1977). An analysis for the magnetization mode for high density magnetic recording. IEEE Trans. Magn..

[21] Hong J, Denver H, Borca-Tasciuc D-A (2006). Fabrication and characterization of nickel nanowire polymer composites. MRS Proc..

[22] Li J, Moskovits M, Haslett T (1998). Nanoscale electroless metal deposition in aligned carbon nanotubes. Chem. Mater..

[23] Matsuoka M, Imanishi S, Hayashi T (1998). Physical properties of electroless Ni-P alloy deposits from a pyrophosphate bath. Plat. and Surf. Finish..

[24] McHenry ME, Majetich SA, Artman JO, DeGraef M, Staley SW (1994). Superparamagnetism in carbon-coated Co particles produced by the Kratschmer carbon arc process. Phys. Rev.. B49: 11 358–11363.

[25] Gong W, Li H, Zhao Z, Chen J (1991). Ultrafine particles of Fe, Co, and Ni: Ferromagnetic metals. J. Appl. Phys..

[26] Abelmann L, Khizroev S, Litvinov D, Bain JA, Zhu J (2000). Micromagnetic simulation of an ultra-small single pole perpendicular write head. J. Appl.. Phys., 87.

[27] Nishikawa M, Kita E, Erata T, Tasaki A (1993). Enhanced magnetization in Co/MgO multilayer thin films. J. Magn. Magn. Mater..

[28] Childress JR, Chien CL (1991). Reentrant magnetic behavior in fcc Co-Cu alloys. Phys. Rev.. B.

[29] Chen JP, Sorensen CM, Klabunde KJ, Hadjipanayis GC (1995). Enhanced magnetization of nanoscale colloidal cobalt particles. Phys. Rev.. B.

