# Correction: Carbon Nanotube Based 3-D Matrix for Enabling Three-Dimensional Nano-Magneto-Electronics

**DOI:** 10.1371/annotation/2275dc86-5426-45ea-ac6f-05ced40182a7

**Published:** 2012-10-04

**Authors:** Jeongmin Hong, Eugenia Stefanescu, Ping Liang, Nikhil Joshi, Song Xue, Dmitri Litvinov, Sakhrat Khizroev

There is an error in the title of the article. The correct title is:

Carbon Nanotube Based 3-D Matrix for Enabling Three-Dimensional Nano-Magneto-Electronics.

The correct citation is: Hong J, Stefanescu E, Liang P, Joshi N, Xue S, et al. (2012) Carbon Nanotube Based 3-D Matrix for Enabling Three-Dimensional Nano-Magneto-Electronics. PLoS ONE 7(7): e40554. doi:10.1371/journal.pone.0040554

